# Development and Evaluation of Thymol Microparticles Using Cellulose Derivatives as Controlled Release Dosage form

**Published:** 2015

**Authors:** Zahra Zamani, Daryoush Alipour, Hamid Reza Moghimi, Seyed Ali Reza Mortazavi, Mostafa Saffary

**Affiliations:** a *Department of Animal Science, Faculty of Agriculture, Bu-Ali Sina University, Hamedan, Iran.*; b *School of Pharmacy, Shahid Beheshti University of Medical Sciences, Tehran, Iran. *; c *Kashan University of Medical Sciences, Kashan, Iran.*

**Keywords:** Thymol, Microparticles, Hydroxypropyl Methylcellulose, Ethylcellulose, Controlled Release

## Abstract

Thymol, an important and advantageous component of many essential oils, has been applied as an antimicrobial agent in animals. To increase the duration of action of this compound in ruminants, it was decided here to prepare a controlled release carrier for thymol.

Hydroxy propyl methyl cellulose (HPMC) and ethyl cellulose (EC) were used as the matrix polymer here. Mixtures of thymol with eight different ratios of these polymers were then prepared using emulsion solvent evaporation method (F_1_ to F_8_). The prepared microparticles were evaluated for production yield, entrapment efficiency, drug content, particle size, drug release behavior, release kinetics (zero order, first order and Fickian matrix diffusion for spheres) and characterized by Fourier transform infrared spectroscopy (FTIR), differential scanning calorimetry (DSC) and scanning electron microscopy (SEM). Mean particle size of microparticles was 1.03 ± 0.02 mm. SEM study revealed that the microparticles were slightly irregular, rough and porous. The formulation with HPMC: EC ratio of 5:1 (F6) showed the highest drug loading (38.8%) and entrapment efficiency (61.2%). This formulation also showed optimum *in-vitro *drug release. The best fit of release kinetics was achieved with Fickian matrix diffusion for spheres (linear amount released vs t^0.43^). The FTIR spectroscopic and DSC studies show possible interaction between drug and polymers.

In this study, thymol was successfully loaded in microparticles prepared from HPMC and EC. These microparticles can be used in further trials to evaluate the effect of slow release thymol on rumen fermentation parameters in ruminants.

## Introduction

The antimicrobial activity of essential oils has been recognized for many years and has formed the basis of many applications. Various anti-bacterial agents have been identified from natural essential oils (1) including thymol (2-isopropyl-5-methylphenol) that has reportedly performed well compared to other antimicrobial agents. Thymol is able to inhibit both Gram-positive and Gram-negative bacteria, including the potential pathogenic strains of *Bacillus subtilis*, *Escherichia coli*, *Klebsiella pneumoniae* and *Staphylococcus aureus* (2). Thymol and thymol-rich essential oils have been beneficially tested in medicine, food flavoring, agricultural, animal nutrition and pest control (3-6). It has been shown that, essential oils can modulate the ruminal fermentation and eventually improvement of nutrient utilization by ruminants through their antimicrobial activities (7).

Studies on the bioavailability and pharmacokinetics of various volatile terpenes, major compounds involved in the antimicrobial activity of essential oils, show that they are rapidly absorbed and metabolized (8). Rapid absorption limits the luminal availability of these compounds for antimicrobial activity. Therefore, it is hypothesized here that their effects on the microflora could be improved by the use of controlled release products (9).

Many studies have been conducted to develop controlled drug delivery systems including tablets, capsules, pellets, patches and gels for oral, bucal, nasal, ocular, and topical drug delivery in human and animal (10). Controlled release drug delivery systems (CRDDS) are aimed to control the rate, time and place of drug release in the body. These systems should be predictable and reproducible with desired rate of drug release (11). CRDDS significantly enhances the therapeutic effect of drugs. Polymers play an integral role in such systems. Natural, semisynthetic and synthetic polymers are increasingly used as drug carriers which play a crucial role in controlled release formulations (12). Cellulose derivatives have been used in many trials as extended and delayed release dosage forms, sustained release matrices, binders in granules and tablets and many other applications (13). These polymers can be degraded in animal rumen as well and, therefore, can make good candidates for controlled drug delivery in ruminants.

The objective of this work was to develop controlled release microparticles of thymol using ethyl cellulose (EC) and hydroxyl propyl methyl cellulose (HPMC) as the matrix and asses the influence of HPMC: EC ratio on the physicochemical properties of the microparticles.

## Experimental

*Materials*

Thymol, Dichloromethane (DCM), Acetone and Tween 80 were obtained from Merck Chemical Company (Germany). Ethyl cellulose (EC, 48% ethoxyl content, = 10 cps) was purchased from Sigma-Aldrich company. Hydroxypropyl methyl cellulose (HPMC, Metolose® 90 SH; = 4000 cps) was obtained from Shin-Etsu Chemical (Japan). Aerosile was obtained from Irandarouk Company (Iran). All other solvents and reagents were of standard analytical and chemical.

*Preparation of microparticles*

Solvent evaporation method (14) was used to prepare thymol microparticles. Firstly, thymol and polymers were weighed and dissolved in a mixture of acetone and DCM (2: 1) to form slurry. Aerosile was then added to the slurry and this combination was then dispersed in aqueous phase (distilled water containing 0.03 % w/v Tween 80) and agitated at 500 rpm for 2 h using a mechanical stirrer (Heidolph, Germany). The microparticles were recovered by filtration, washed with distilled water and air dried overnight at room temprature. To optimize the loading and release behavior of the particles, 8 formulations (F_1_-F_8_) were prepared using different ratio of HPMC and EC (Table 1).

**Table 1:** Formulation of thymol microparticles

**Formulation code**	**Thymol** **(mg)**		**HPMC (mg)**	**EC (mg)**	**HPMC:EC**	**Drug:polymer**	**Tween80 (mg)**	**Aerosile (mg)**
F_1_		400	200	200	1:1	1: 1	45	50
F_2_		400	266	133	2:1	1: 1	45	50
F_3_		400	287	115	2.5:1	1: 1	45	50
F_4_		400	300	100	3:1	1: 1	45	50
F_5_		400	320	80	4:1	1: 1	45	50
F_6_		400	334	66	5:1	1: 1	45	50
F_7_		400	343	57	6:1	1: 1	45	50
F_8_		400	350	50	7:1	1: 1	45	50

*Particle size determination*

Size distribution of microparticles was studied by optical microscopy using zeiss microscope. A small quantity of microparticles was dispersed on the slide and the diameters were measured at 100 magnification. An average of 100 particles was measured for each formulation.

*FTIR Spectroscopy*

FTIR studies were performed to investigate any possible interaction between thymol and of formulation components. FTIR measurement was performed in the absorbance mode, using WQF-510 Fourier Transform Spectrometer (Rayleigh Optics, China) equipped with a KBr beam splitter and a DLa TGS (deuterated lanthanide triglycine sulphate) detector and µmax microscope (PIKE, USA). The spectra were scanned in the mid-IR range from 400 to 4000 cm^-1^with a resolution of 4 cm^-1^. Around one hundred scans were coded for each spectrum and the spectra were normalized against the background spectrum.

*Percentage yield*

The dried microparticles were weighed and percentage yield of prepared microparticles was calculated by following formula (15).

Percentage yield = (weight of harvested particles/weight of initial material) × 100

*Drug Loading and Drug Entrapment Efficiency*

To determine the drug entrapment efficiency (DEE) and drug loading (DL), the microparticles were suspended in 20 ml ethanol 75% and stored at room temperature to dissolve. The solution was sonicated for 6 minutes and then filtered through Whatman filter paper and filtrate was analyzed for drug content. The drug entrapment efficiency and drug loading were calculated as follows (16):

DL = (weight of drug in microparticles/total microparticles weight) × 100

DEE = (actual weight of drug in sample/theoretical weight of drug) × 100.

*Drug determination*

Thymol measurements were carried out by UV spectrophotometry at 276nm using CECIL UV/Vis spectrophotometer model CE2021 (UK) equipped with 1cm thickness quartz cells.

Thymol, HPMC, EC and Aerosile Solutions in ethanol 75% (all 10 μg ml^-1^) were prepared and scanned in the range of 200-400 nm against ethanol 75% as the reference. Maximum absorbance of thymol was observed at 276 nm. Maximum absorbance of HPMC and EC were observed at 268 & 269nm respectively. Aerosile do not show any absorbance in UV range (200-400 nm). To prevent interaction from formulation ingredients, all thymol measurements were against a blank containing the formulation ingredients.

*Drug Release Study*

Drug release study was performed using USP Dissolution Testing Apparatus (Basket type) (Erweka, DT820, Germany) at 37 ± 0.5°C and at 75 rpm using 500 mL phosphate buffer (pH 6.8) as a dissolution medium (n = 4). Microparticles equivalent to 38 mg of thymol were used for the test. Five milliliters of sample solution was withdrawn at predetermined time intervals, centrifuged at 2000 rpm for 5 min and analyzed spectrophotometrically. An equal amount of fresh dissolution medium was replaced immediately after sampling.

*Differential Scanning Calorimetry (DSC)*

DSC analysis was performed using Shimadzo DSC (DSC-60 Kypto, Japan). The instrument was calibrated with indium (calibration standard purity > 99.999%) for melting point. A heating rate of 10°C/min was employed in the range of 5-95°C. Analysis was performed under a nitrogen purge (50 ml/min) using standard aluminum pan and about 10 mg sample. An empty pan was used as reference.

*Scanning Electron Microscopy*

SEM analysis was carried out using a scanning electron microscope (Tescan Vega TS 5136 MM). Prior to examination, samples were mounted on an aluminum stub using a double sided adhesive tape and making it electrically conductive by coating with a thin layer of gold (approximately 20 nm) in vacuum. The scanning electron microscope was operated at an acceleration voltage of 30KV and resolution of 4000.

*Kinetic of release*

In order to study the release kinetics of micoparticle, data obtained from *in vitro *drug release studies were fitted into different kinetic mathematical models. These models were as follows: 

1 *Q*_t_* = k*_0_* t *(zero-order equation)

2 *ln Q*_t_* = ln Q*_0_* - k*_1_*. t *(first-order equation)

3 *Q*_t_* = k*_f _*t*^0.4 3^(Fickian matrix diffusion for spheres)

Where *Q *is the cumulative amount of drug released at time *t*, *Q*_0 _is the initial amount of drug in the microparticles. *K*_0_*, k*_1_ and *k*_f _are rate constants of zero order, first order and Fickian equations respectively (17).

*Stability study*

Stability study was carried out at room temperature for 90 days. The selected microparticles were packed in glass containers and closed with air tight closure and stored for 90 days. Samples were analyzed at the end of 90 days and evaluated for drug content, percentage of drug entrapment efficiency and *in vitro *drug release studies.

*Statistical Analysis*

One way analysis of variance (ANOVA) was performed on the data. P-values less than 0.05 were considered as significant. All statistical calculations were performed using SAS software (Version 7) (18). All data have as mean SD (n = 3).

## Results and Discussions

*Characterization of prepared formulations*

Table 2 provide loading, entrapment efficiency, particle size and yield of different formulations prepared here. The range of particle size was from 0.457 ± 0.022 mm to 1.577 ± 0.007 mm. Higher proportion of HPMC resulted in a larger size of microparticles which can be due to higher viscosity of the solution and decrease in stirring efficiency (19). In the higher viscosity of the internal phase, the greater quantity of energy needed to break the drug-polymer droplets into smaller particles (20).

As shown in Table 2, DEE and DL were affected by HPMC: EC ratio in the following orders F_6 _> F_5 _> F_4 _> F_7 _> F_3 _> F_8 _> F_2 _> F_1_ and F_6 _> F_5 _> F_3 _> F_7 _> F_4 _> F_1 _> F_8_ (for DEE and DL, respectively). By increasing the amount of HPMC in F_8_, a slight decrease in percentage of entrapment efficiency and drug loading was observed. This could be due to the formation of weak network that allows leaching out of more particles during preparation (21). The percentage yield was found to be in the ranges of 66.8 to 76.16 % and the yield was found satisfactory in all formulations as reported in Table 2.

**Figure 1:**
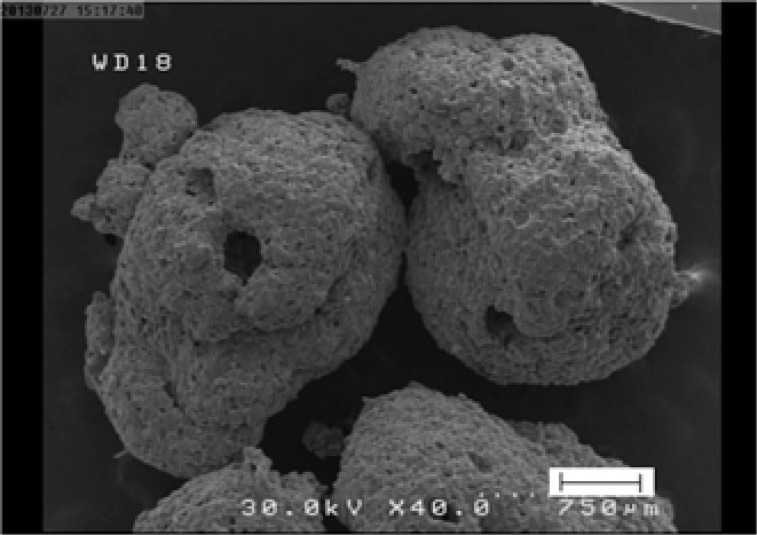
SEM micrograph of selected microparticles (F_6_).

Microparticles of this study were slightly irregular with rough surfaces as revealed by SEM images (Figure 1). The pores at the surface of microparticles may be because of the rapid evaporation of solvent as has been reported by Dandge and Dehghan (15). It is speculated that at earlier stage of solvent evaporation process a crust is formed on the surface of the droplets which prevents the evaporation of the solvent. This in turn causes the building up of the vapor pressure resulting in small eruptions and formation of openings. Distinct pores are evident on the surface of microparticles which will be partly responsible for the drug release as it has been suggested by some previous investigators (15).

**Table 2:** Loading proportion, yield and particle size of microparticles prepared in the present investigation*.

**Formulation codes**	**Loading %**	**Drug entrapment** **efficiency ( %)**	**Particle size (mm)**	**Yield (%)**
F_1_	34.69^ab^***1.07	43.94^c^1.36	0.529^d^ 0.031	66.810.28
F_2_	30.21^c^0.99	45.52^c^1.49	0.457^e^0.022	74.774.81
F_3_	38.43^a^0.47	56.18^ab^0.69	0.869^c^0.017	69.140.23
F_4_	34.99^ab^1.97	57.61^a^2.42	0.576^d^0.04	74.20.11
F_5_	38.54^a^2.19	58.34^a^4.08	1.331^b^0.0005	71.120.17
F_6_	38.82^a^0.99	61.22^a^3.39	1.574^a^0.011	76.162.84
F_7_	35.65^ab^0.02	57.39^a^0.04	1.577^a^0.007	75.190.81
F_8_	33.91^bc^2.19	49.59^bc^3.21	1.314^b^0.035	71.623.96
**P-value	<0.0001	<0.0001	<0.0001	0.4688

* Data are means ± SD (n = 3).

**P-value in one-way ANOVA.

*** Means within a column with different superscript letters are different (*P *0.05).

*Release studies*

**Figure 2:**
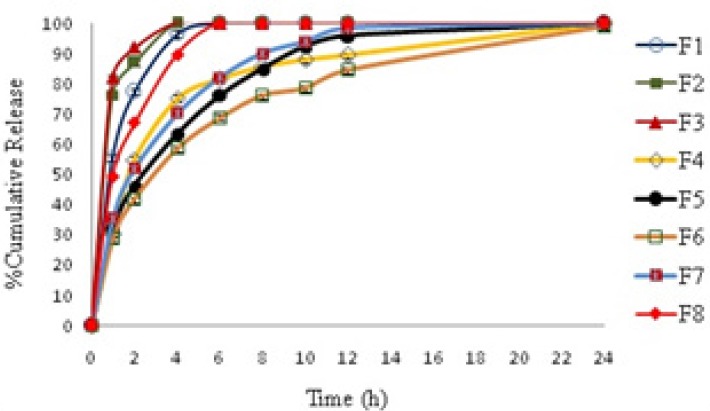
Thymol release profile from microparticles prepared by the solvent evaporation method. See Table 3 for details

Cumulative amount of thymol released from formulations are provided in Table 3 and Figure 2 shows the profiles of drug release against time. As shown in Figure 2, four formulations of F_1_, F_2_, F_3_ and F_8_ release most of their cargo in less than two hours and the entire drug is released from these systems in less than 4 hours. Others formulations, that contain 35.1-40.13% HPMC, show a sustained release behavior over 24 h. Among this formulation F_6_ that showed the slowest release rate was chosen for further studies.

**Figure 3:**
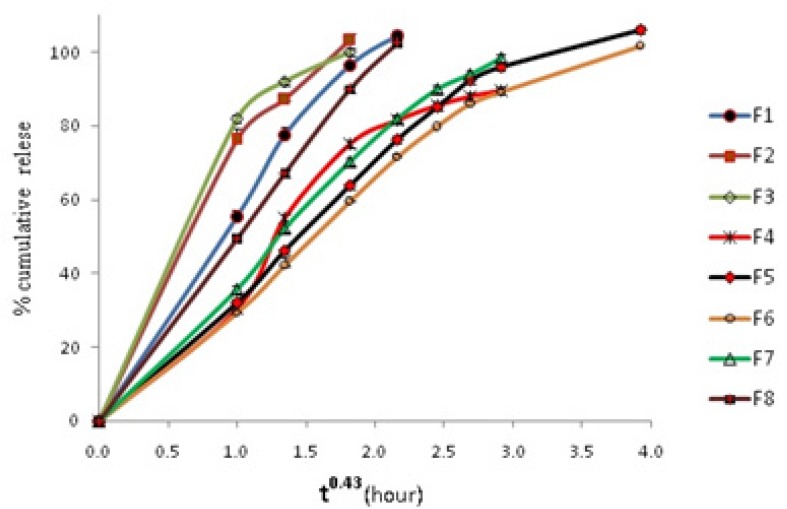
Cumulative amount thymol release from different microparticle formulations vs t^0.43^

The *in-vitro *drug release data of formulations was fitted to first order zero order and Fickian release kinetics for spheres (t^0.43^) as shown in Table 4. Results showed that a Fickian release for spheres is obeyed in all systems. This shows that all systems obey matrix diffusion mechanism for drug release. Figure 3 shows cumulative release vs t^0.43 ^graphs.

**Table 3:** Cumulative amount of thymol release from different formulations*.

**Formulation**	**F**_1_	**F**_2_	**F**_3_	**F**_4_	**F**_5_	**F**_6_	**F**_7_	**F**_8_	**p-value** **
**Time (h)**									
1	53.61^c^***2.07	74.95^b^3.28	83.27^a^3.84	31.65^d^2.24	32.22^d^0.31	28.8^d^2.71	35.18^d^1.72	49.79^c^0.56	<0.0001
2	71.72^b^2.13	86.33^a^1.83	91.89^a^3.61	56.21^d^3.38	46.38^dc^0.99	41.68^d^2.47	48.59d^c^5.23	66.93^b^5.89	<0.0001
4	93.33b^c^1.41	101.26^ab^2.17	109^a^2.93	75.43d1.79	63.22^ef^0.52	55.61^f^8.34	69.6d^e^1.82	88.90^c^1.97	<0.0001
6	100.91^a^1.15	-	-	80.59^b^0.61	75.39^c^1.05	68.79^d^3.61	80.74^b^0.39	100.44^a^2.03	<0.0001
8	-	-	-	84.07^a^1.08	82.7a 0.57	75.96^b^4.64	87.88^a^0.81	-	0.0022
10	-	-	-	85.94^a^b0.10	89.59^a^0.005	77.95^b^6.19	92.35^a^1.77	-	0.0030
12	-	-	-	86.74^b^c0.46	93.10^ab^1.2	83.12^c^4.68	97.65^a^3.17	-	0.0012
24	-	-	-	-	101.96^a^0.84	98.96^b^1.03	-	-	0.0175

* Data are means ± SD (n = 3).

** P-value in one-way ANOVA

*** Means within a row with different superscript letters are different (*P *< 0.05).

**Table 4:** Release constants (K) and correlation coefficients (R) for linear relationship of microparticles for different kinetic models

**Formulation**		**F**_1_	**F**_2_	**F**_3_	**F**_4_	**F**_5_	**F**_6_	**F**_7_	**F**_8_
**Kinetic**									
Zero order	K_0_	9.411	8.818	5.780	4.62	2.986	2.773	5.399	10.38
	R^2^	0.902	0.994	0.937	0.749	0.732	0.787	0.896	0.962
									
First order	K_1_	0.051	0.042	0.027	0.033	0.019	0.019	0.035	0.060
	R^2^	0.850	0.986	0.923	0.648	0.613	0.644	0.811	0.919
									
Fickian	K_f_	42.25	33.01	22.05	29.17	26.57	24.2	32.82	45.86
	R^2^	0.965	0.999	0.978	0.879	0.921	0.951	0.976	0.995

*Differential Scanning Calorimetry measurements*

**Figure 4:**
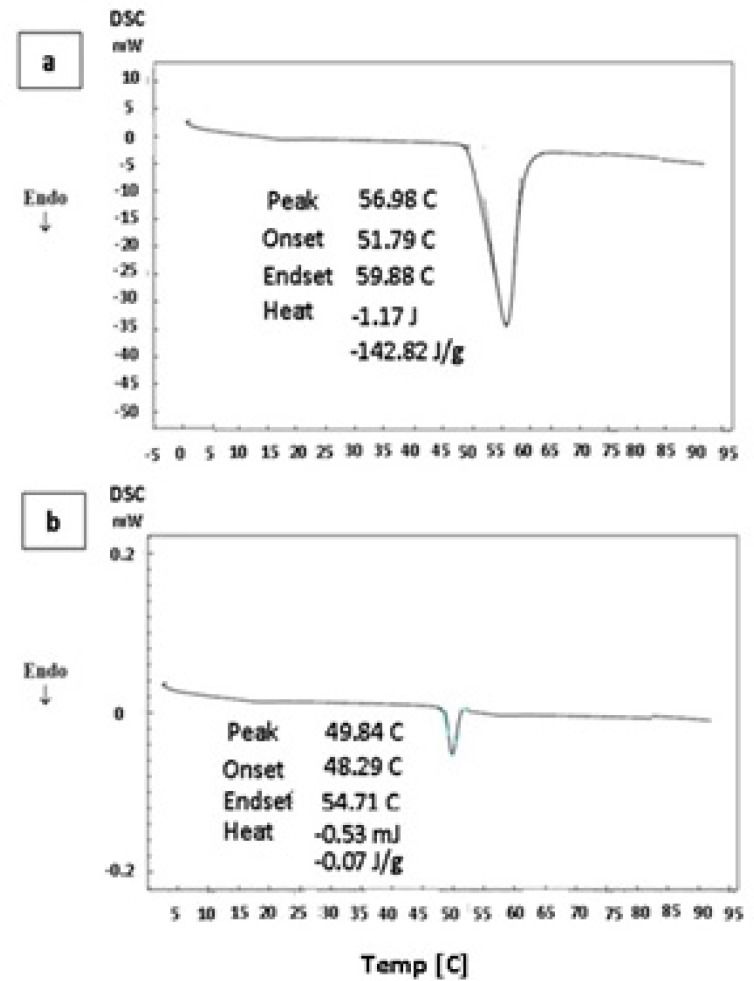
DSC thermograph of thymol (a) and thymol loaded microparticles (b).

DSC thermograms of pure thymol and thymol-loaded microparticles are shown in Figure 4. Pure thymol (Figure 4a) shows an endothermic peak with onset temperature about 51.8°C, which is in good agreement with thymol melting point of 52°C (6). DSC profile of drug-loaded polymer also showed a transition temperature close to thymol melting point (Figure 4b). A similar observation was reported by Wattanasatcha et al (6) for DSC analysis of thymol encapsulated within ethylcellulose/methylcellulose nanospheres. Our DSC studies showed a much lower enthalpy in microsphese in comparison to pure thymol (Figure 4b). It appears that there is a significant reduction of drug crystallinity in the microparticles and might indicate dispersion of thymol molecules in the system. Polymers do not show any transition over the temperature range used here.

*FTIR spectrum analysis*

**Figure 5:**
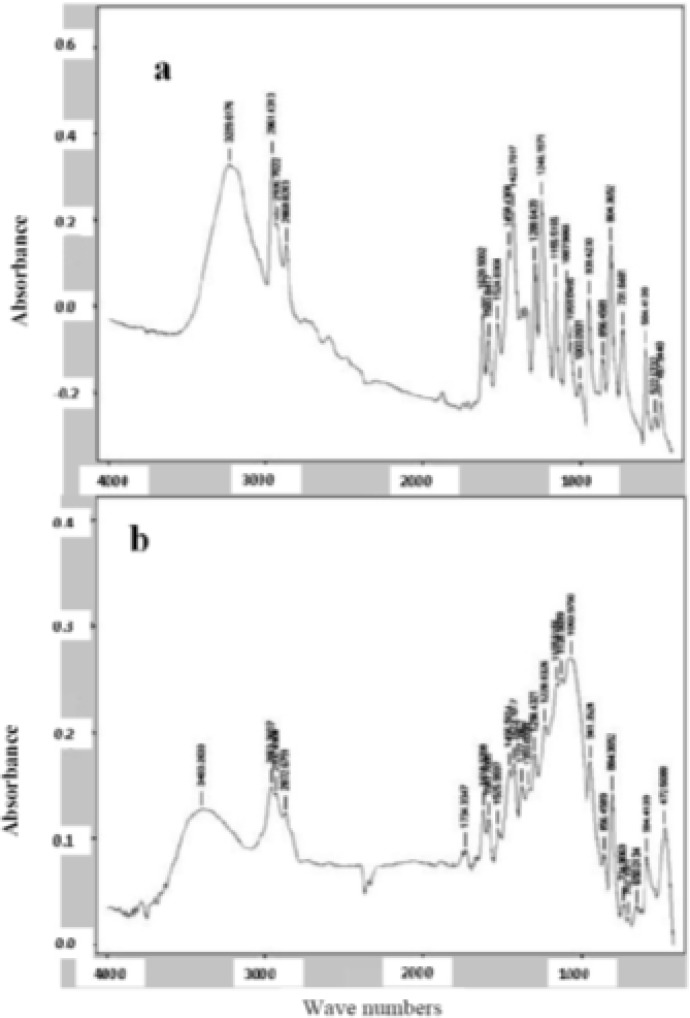
FTIR spectra of thymol (a) and thymol loaded microparticles (b).

FTIR spectra are shown in Figure 5. Thymol spectra (Figure 5a) show a band at 3229 cm^-1^ corresponding to phenolic -OH stretching involving hydrogen bonding. Aromatic character of thymol is exhibited by C = C stretching of benzene ring at 1620 cm^-1^, respectively. In thymol-loaded polymer, the main change was observed for –OH stretching peak (200 shifts) that might indicate interaction of thymol with polymers (Figure 5b).

*Stability study*

**Figure 6:**
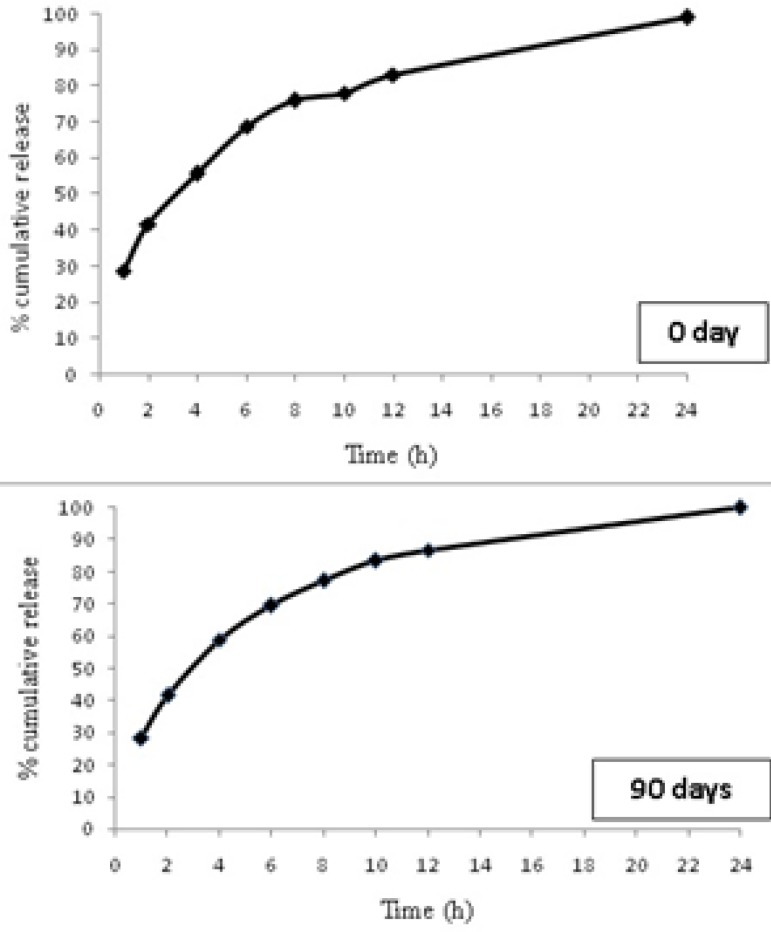
Cumulative amount thymol released from selected microparticle after stability

The 90 days stability studies showed no change in the appearance of the microparticles indicating that the selected formulation was stable during the storage. No significant change in drug content was observed for F6 stored at room temperature (Table 5). *In vitro *drug release studies for F6 formulation were carried out at the end of 90 days and did not reveal any significant change in drug release from selected formulation (Figure 6).

**Table 5:** Stability data of selected microparticles stored at room temperature for 90 days.*

Characteristics	Time in days	
	0 day	90 days
% DL	38.820.99	38.440.36
%DEE	61.223.39	61.010.58

* Data are means ± SD (n = 3).

study (90 days) in comparison to the initial profile.

## Conclusion

In this study thymol was successfully loaded in microparticles made from HPMC and EC and was slowly released in an *in vitro *release study. Both polymers showed a significant effect on drug entrapment efficiency, drug loading, particle size and drug release behavior. Considering all the properties evaluated here, formulation F_6_ (HPMC: EC ratio of 5: 1), was found to be the best microparticle formulation. Microparticle formulation F_6_ showed better controlled effect over 24 hours than other formulations. Microparticle showed a spherical shape with somewhat rough surfaces and size of 1.03 mm, which can be considered suitable for in vivo trial in ruminants. Further in vivo researches are in progress in our laboratories to investigate the antimicrobial effect of these microparicles in the gastrointestinal tract of sheep.
